# Golgi Reassembly and Stacking Protein (GRASP) Participates in Vesicle-Mediated RNA Export in *Cryptococcus neoformans*

**DOI:** 10.3390/genes9080400

**Published:** 2018-08-08

**Authors:** Roberta Peres da Silva, Sharon de Toledo Martins, Juliana Rizzo, Flavia C. G. dos Reis, Luna S. Joffe, Marilene Vainstein, Livia Kmetzsch, Débora L. Oliveira, Rosana Puccia, Samuel Goldenberg, Marcio L. Rodrigues, Lysangela R. Alves

**Affiliations:** 1 Departamento de Microbiologia, Imunologia e Parasitologia da Escola Paulista de Medicina-UNIFESP, São Paulo, SP 04023-062, Brazil; roberta.peresdasilva@nottingham.ac.uk (R.P.d.S.); ropuccia@gmail.com (R.P.); 2 School of Life Sciences, University of Nottingham, Nottingham NG7 2RD, UK; 3 Instituto Carlos Chagas, Fundação Oswaldo Cruz, Fiocruz-PR, Curitiba, PR 81310-020, Brazil; sdt.martins@gmail.com (S.d.T.M.); flaviar23@gmail.com (F.C.G.d.R.); sgoldenb@fiocruz.br (S.G.); marciolrodrig@gmail.com (M.L.R.); 4 Instituto de Microbiologia Professor Paulo de Góes, Universidade Federal do Rio de Janeiro, Rio de Janeiro, RJ 21941-901, Brazil; juju.rizzo@gmail.com; 5 Centro de Desenvolvimento Tecnológico em Saúde (CDTS), Fundação Oswaldo Cruz, Rio de Janeiro, RJ 21040-900, Brazil; lujoffe@gmail.com (L.S.J.); debora_leite@yahoo.com.br (D.L.O.); 6 Centro de Biotecnologia e Departamento de Biologia Molecular e Biotecnologia, Universidade Federal do Rio Grande do Sul, Porto Alegre, RS 91501-970, Brazil; mhv@cbiot.ufrgs.br (M.V.); liviak@cbiot.ufrgs.br (L.K.)

**Keywords:** *Cryptococus neoformans*, RNA, extracellular vesicles, GRASP, Atg7, unconventional secretory pathway

## Abstract

Golgi reassembly and stacking protein (GRASP) is required for polysaccharide secretion and virulence in *Cryptococcus neoformans*. In fungal species, extracellular vesicles (EVs) participate in the export of polysaccharides, proteins and RNA. In the present work, we investigated if EV-mediated RNA export is functionally connected with GRASP in *C. neoformans* using a *grasp*Δ mutant. Since GRASP-mediated unconventional secretion involves autophagosome formation in yeast, we included the *atg7*Δ mutant with defective autophagic mechanisms in our analysis. All fungal strains exported EVs but deletion of *GRASP* or *ATG7* profoundly affected vesicular dimensions. The mRNA content of the *grasp*Δ EVs differed substantially from that of the other two strains. The transcripts associated to the endoplasmic reticulum were highly abundant transcripts in *grasp*Δ EVs. Among non-coding RNAs (ncRNAs), tRNA fragments were the most abundant in both mutant EVs but *grasp*Δ EVs alone concentrated 22 exclusive sequences. In general, our results showed that the EV RNA content from *atg7*Δ and WT were more related than the RNA content of *grasp*Δ, suggesting that GRASP, but not the autophagy regulator Atg7, is involved in the EV export of RNA. This is a previously unknown function for a key regulator of unconventional secretion in eukaryotic cells.

## 1. Introduction

Extracellular vesicle (EV) formation and release constitute a ubiquitous export mechanism of proteins, DNA and RNA [1,2]. EVs play key roles in processes of cell communication, homeostasis, immunopathogenesis and microbial virulence [1,2]. EV formation is a conserved mechanism in both prokaryotic and eukaryotic cells [3]. In fungi, EVs participate in the transport of macromolecules across the cell wall [4,5,6]. Fungal EVs transport a variety of macromolecules including proteins, lipids, glycans, pigments and, as more recently described, RNA [4,6,7,8,9].

EV biogenesis in fungi is still poorly understood. It has been hypothesized that EV biogenesis in eukaryotes is a complex process that is regulated at multiple levels [10,11]. EV formation is part of the unconventional secretion machinery in eukaryotes and general regulators of unconventional secretion have been identified. GRASP (Golgi reassembly and stacking protein) is a secretion regulator originally characterized in human cells as part of the Golgi cisternae stacking and ribbon formation [12,13]. During stress, GRASP is required for protein delivery to the plasma membrane or to the extracellular space by an unconventional pathway that involves autophagosome-like structures [14]. In mammalian cells, GRASP is also involved in the delivery of a mutant form of cystic fibrosis transmembrane conductance regulator to the plasma membrane in a Golgi-independent manner [15]. In *Drosophila melanogaster*, GRASP participates in the delivery of integrins from the ER directly to the plasma membrane, thus bypassing the Golgi [16]. In the amoeba *Dictyostelium discoideum*, a GRASP orthologue (GrpA) was necessary for acyl-coenzyme A-binding protein (AcbA) secretion during spore differentiation [17]. In the yeast species *Saccharomyces cerevisiae* and *Pichia pastoris*, another GRASP orthologous (Ghr1) was also required for starvation-induced secretion of AcbA [18,19].

In the yeast-like neuropathogen *Cryptococcus neoformans*, GRASP was required for polysaccharide export to the extracellular space. Polysaccharide secretion is fundamental for virulence in *C. neoformans* [20] and, in fact, a *grasp*Δ mutant was hypovirulent in mice [20]. Polysaccharide export in *C. neoformans* is mediated by EVs but connections between GRASP functions and EV cargo remain uncharacterized.

Autophagy is a self-degradative process conserved in eukaryotes, presenting a housekeeping role by degrading dysfunctional components such as organelles and misfolded proteins [21]. The Atg7 is an autophagy regulator protein member of the ubiquitin-activating enzyme (E1) family involved in this process [22]. The Atg proteins have non-canonical roles in distinct cellular pathways. For example, *Toxoplasma gondii* Atg8 localizes to the apicoplast and is essential for organelle homeostasis and survival of the tachyzoite stage of the parasite [23]. Atg7 non-autophagic roles include cathepsin K secretion in bone osteoclasts [24], IFNγ-mediated antiviral activity against virus replication [25], adipogenesis in mice [26] and cell cycle regulation via p53 interaction and expression of p21 in mouse embryonic fibroblasts [27].

Autophagy regulators play key roles in cryptococcal physiology and, in fact, we have recently demonstrated that the putative autophagy regulator Atg7 affects both physiological and pathogenic mechanisms in *C. neoformans* [28]. In *D. discoideum*, GRASP-mediated unconventional secretion is mediated by autophagosomes, showing that there is a connection between these processes [18,29].

The role of unconventional secretion regulators in vesicular export of RNA is unknown but the functional connections between GRASP and Atg7 led us to evaluate whether these proteins affected extracellular RNA export in *C. neoformans*. Our results suggest that GRASP, but not Atg7, is a key regulator of vesicular export of RNA in *C. neoformans*.

## 2. Material and Methods

### 2.1. Fungal Strains and Growth Conditions

The *C. neoformans* strains used in this study included the parental isolate H99 and the mutant strains *atg7*Δ and *grasp*Δ, which were generated in previous studies by our group [20,28]. Fungal cultures were maintained at 30 °C in Sabouraud dextrose plates (1% dextrose, 4% peptone). Cells recovered from the stationary cultures were used to inoculate minimal medium composed of dextrose (15 mM), MgSO_4_ (10 mM), KH_2_PO_4_ (29.4 mM), glycine (13 mM) and thiamine-HCl (3 µM) for further cultivation for three days at 30 °C, with shaking. All protocols adhered to the biosecurity demands of the Carlos Chagas Institute of Fiocruz (Curitiba, Brazil).

### 2.2. Extracellular Vesicle Isolation and Diameter Determination

EVs were isolated from fungal culture supernatants as previously described [4]. Briefly, cell-free culture supernatants were recovered by centrifugation at 4000× *g* for 15 min at 4 °C and the resulting supernatants were pelleted at 15,000× *g* for 30 min to remove small debris. The final supernatants were concentrated by a factor of 20 in an Amicon ultrafiltration system (100-kDa cutoff, Millipore, Burlington, VT, USA). Concentrated supernatants were centrifuged at 15,000× *g* for 30 min to ensure the removal of aggregates and the resulting supernatant was then ultracentrifuged at 100,000× *g* for 1 h to precipitate vesicles. Vesicle pellets were washed once in phosphate-buffered saline (PBS) and the final pellets were suspended in PBS. For analysis of EV dimensions, nanoparticle tracking analysis (NTA) was performed on a LM10 Nanoparticle Analysis System, coupled with a 488 nm laser and equipped with a _S_CMOS camera and a syringe pump (Malvern Panalytical, Malvern, UK). The data was acquired and analyzed using the NTA 3.0 Software (Malvern Panalytical). EVs from all samples were diluted 1:30 in filtered PBS (0.22 µM) and measured within the optimal dilution range previously described by Maas and colleagues (9 × 10^7^–2.9 × 10^9^ particles/mL) [30]. Polystyrene microspheres (100 nm) were used for equipment calibration. Samples were injected using a syringe pump speed of 50 and three videos of 60 s were captured per sample, with the camera level set to 15, gain set to 3 and viscosity set to water (0.954–0.955 cP). For data analysis, the gain was set to 10 and detection threshold was set to 5 for all samples. Levels of blur and max jump distance were automatically set. Particle detection values were normalized to the total number of cells in cultures from which each sample was obtained.

### 2.3. Small RNA Isolation

Small RNA (sRNA)-enriched fractions were isolated with the miRNeasy mini kit (Qiagen, Hilden, Germany) and then treated with the RNeasy MinElute Cleanup Kit (Qiagen), according to the manufacturer’s protocol, to obtain small RNA-enriched fractions. The success of the sRNA extraction was assessed in representative EV preparations that were treated with 30 U DNase I (Qiagen) and characterized in an Agilent 2100 Bioanalyzer (Agilent Technologies, Santa Clara, CA, USA). To confirm that the RNA was confined within the EVs, vesicle samples were treated with 0.4 μg μl^−1^ RNase (Promega, Madison, WY, USA) for 10 min at 37 °C before RNA extraction, as previously described [9].

### 2.4. RNA Sequencing

One hundred ng of purified sRNA were used for RNA-seq analysis from two independent biological replicates. The RNA-seq was performed in a SOLiD 3 plus platform using the RNA-Seq kit (Life Technologies, Carlsbad, CA, USA) according to the manufacturer’s recommendations.

### 2.5. Cellular RNA Isolation and Quantitative PCR

Yeast cells were grown in minimal medium for 72 h, pelleted by 1 min centrifugation at 14.000× *g*, washed in PBS, suspended in the lysis buffer provided in miRCURY™ RNA Isolation Kit–Cell & Plant (Exiqon, Vedbaek, Danmark) and vortexed 5 times in acid washed glass beads (425–600 micron, Sigma-Aldrich, St. Louis, MO, USA). The lysate was centrifuged for 2 min at 14.000× *g* and the supernatants were collected for RNA isolation with the mirCURY™ kit, following the manufacturer’s instructions. The RNAs were eluted in ultrapure water and treated with RQ1 RNase-Free Dnase (Promega) following the manufacturer’s instructions. Reverse transcription reactions with the DNAse-treated RNAs were performed with a random primer and the ImProm-II™ Reverse Transcription System (Promega), following the manufacturer’s instructions. Real time PCR reactions were performed using SYBR^®^ Select Master Mix and run and analyzed using the LightCycler^®^ 96 System (Roche, Basel, Switzerland). The primers corresponded to CNAG_03103 Cullin3 Forward GCCATACGGGAGATACAGAAC, Reverse GAGGTGTTGGACGATGAGAG, CNAG_07590 V_typeH Forward TCATGCTCAACGAAGTCAGG, Reverse GGAAGCAGTGGTTGTGAATG, CNAG_03337 hypothetic Forward CGGTCTTTATCGCTGCTGTAT, Reverse ATTGAAGAGTGGATGTCGTGG and CNAG_00483 Actin Forward CCACACTGTCCCCATTTACGA, Reverse CAGCAAGATCGATACGGAGGAT Each reaction was performed using 10 ng of cDNA. The experiment was performed in triplicates and the expression levels relative to actin were calculated according to Pfaffl’s method using *t*-test for the statistical analysis [31].

### 2.6. In Silico Data Analysis

The sequencing data were analyzed using the version 9.1 of CLC Genomics Workbench©. The reads were trimmed on the basis of quality, with a threshold Phred score of 15. The reference genomes used for mapping were obtained from the NCBI database (*C. neoformans*-GCA_000149245.3). The alignment was performed as follows: additional 100-base upstream and downstream sequences; 10 minimum number of reads; 2 maximum number of mismatches; −2 nonspecific match limit and minimum fraction length of 0.9 for the genome mapping or 1.0 for the RNA mapping. The minimum reads similarity mapped on the reference genome was 80%. Only uniquely mapped reads were considered in the analysis. The libraries were normalized per million and the expression values for the transcripts were recorded in RPKM (reads per kilobase per million), we also analyzed the other expression values-TPM (transcripts per million) and CPM (counts per million).

### 2.7. Data Access

The data is deposited to the Sequence Read Archive (SRA) database of NCBI (Bethesda, MA, USA) under study accession number (SRA: SRX2793565 to 67).

## 3. Results

### 3.1. Lack of GRASP Results in Changes in the RNA Content of Cryptococcus neoformans Extracellular Vesicles

Our experimental model included wild type (WT) and two mutant strains of *C. neoformans*. WT cells corresponded to strain H99, a standard and widely investigated clinical isolate. Knockout mutant strains (KO) lacked expression of two regulators of cryptococcal pathogenicity, *GRASP* and *ATG7* [20,28].

**Figure 1:**
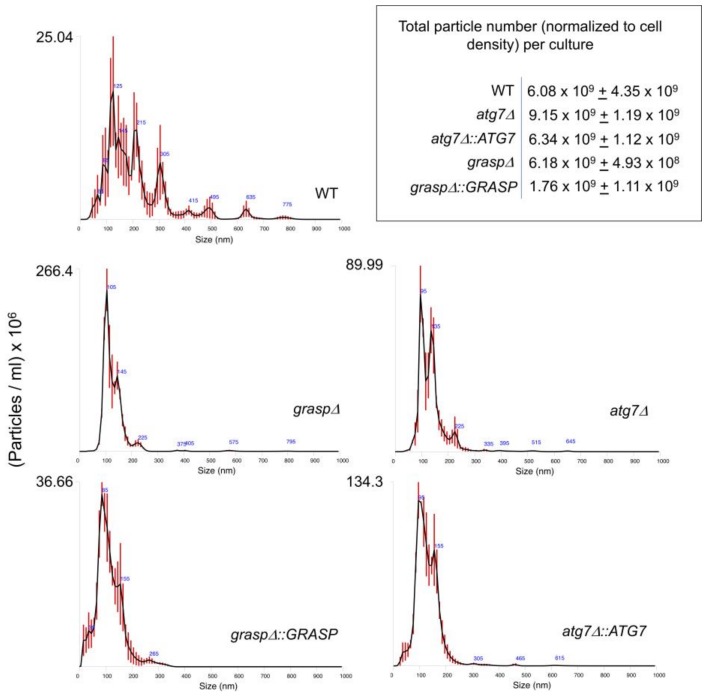
Nanoparticle tracking analysis of *Cryptococcus neoformans* extracellular vesicles (EVs) comparing wild type (WT), mutant (*grasp*Δ and *atg7*Δ) and complemented (*grasp*Δ::*GRASP* and *atg7*Δ::*ATG7*) cells. Results are representative of two independent biological replicates producing similar profiles. Particles were quantified in EV samples suspended in 150 mL phosphate-buffered saline (PBS). Particle detection values shown in the upper, right panel were normalized to the total number of cells in the cultures from which each sample was obtained.

We first asked whether the lack of either *GRASP* (*grasp*Δ) or *ATG7* (*atg7*Δ) expression would affect the EVs composition. The analysis of diameter distribution of wild type EVs by nanoparticle tracking analysis (Figure 1) revealed a major population of cryptococcal vesicles in the 50–250 nm range. Peaks of EVs corresponding to approximately 300, 410, 500 and 630 nm were also observed. Although the dimensions of cryptococcal EVs have been traditionally determined by dynamic light scattering and/or electron microscopy, the results obtained by nanoparticle tracking analysis were consistent with the previous literature [32]. Deletion of *GRASP* or *ATG7* produced a clear impact on the size distribution of cryptococcal EVs. In comparison to WT cells, peaks corresponding to sizes higher than 300 nm were no longer observed. A minor peak at 225 nm and major, sharp peaks at 100 and 140 nm were observed in EVs produced by both mutants. Complementation of mutant cells resulted in EV fractions enriched in the 100–300 nm range, but the minor peaks at 415, 500 and 600 nm observed in WT cells were still not detectable. Although deletion of *GRASP* or *ATG7* resulted in modified EV detection, no statistical differences were observed between the different samples. In summary, the nanoparticle tracking analysis revealed that deletion of *GRASP* and *ATG7* affected EV properties in *C. neoformans*. We then asked whether the differences in EV diameters correlated with the RNA content in *C. neoformans* EVs.

Total RNA was isolated from fungal EVs and two independent biological replicates were subjected to RNA-seq (Figure S1). In order to compare the EV-RNA composition between the knockout (*atg7*Δ and *grasp*Δ) and the WT strains we first aligned the RNA-seq reads with the *C. neoformans* H99 genome (GCA_000149245.3) sequences. We used the raw data available for isolate H99 from our previous work [9] and compared them with the *atg7*Δ and *grasp*Δ EV RNA (Table 1). For all *C. neoformans* strains about 85% of the EV-RNA reads mapped to intronic regions, while less than 10% mapped to exons. A similar profile was observed for the *C. neoformans* WT strain (H99) in our previous work [9].

**Figure 2:**
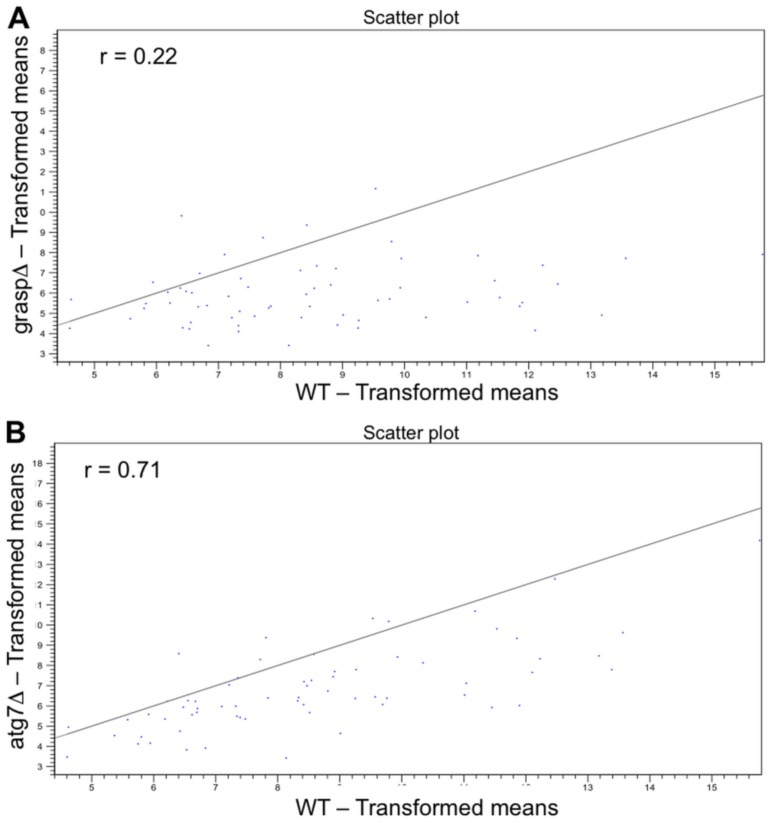
Correlation between the EV-mRNA sequences of *grasp*Δ vs. WT samples (**A**) and *atg7*Δ vs. WT preparations (**B**). The transformed mean read values for WT EVs are in the X-axis, while those obtained from mutant vesicles are in the *y*-axis.

Analysis of EV-mRNAs showed that the correlation between WT and *atg7*Δ (r 0.71) sequences was greater than that for WT and *grasp*Δ (r 0.22) (Figure 2A,B). This result indicates that the mRNA content in WT EVs was closer to that of *atg7*Δ vesicles than to the content of *grasp*Δ EVs.

**Figure 3:**
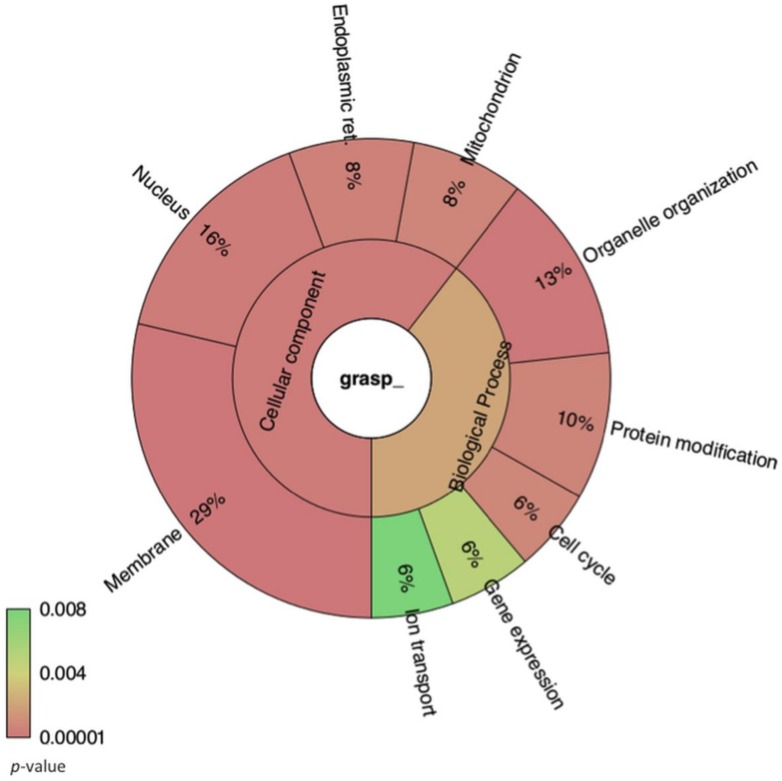
Krona chart representing the gene ontology of mRNA sequences enriched in EVs isolated from the *C. neoformans grasp*Δ mutant. The percentage refers to the relative enrichment for the Gene Ontology (GO) terms. The colors represent the *p*-value for each term plotted in the chart.

**Figure 4:**
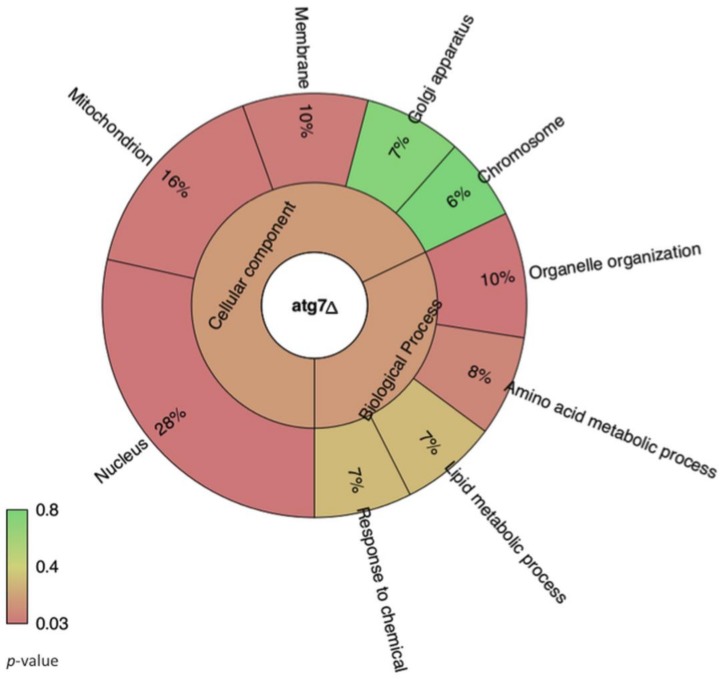
Krona chart representing the gene ontology of mRNA sequences enriched in EVs isolated from the *C. neoformans atg7*Δ mutant. The percentage refers to the relative enrichment for the GO terms. The colors represent the *p*-value for each term plotted in the chart.

We next performed paired comparisons (WT versus *grasp*Δ and WT versus *atg7*Δ) and applied the statistical negative binomial test [33] and the filters RPKM ≥ 50, log2 ≥ 2 and false discovery rate (FDR) ≤ 0.01. From the WT versus *grasp*Δ analysis, 266 mRNAs were identified as enriched in the EVs from the *grasp*Δ mutant (Table S1). From these transcripts, we observed enrichment in cellular components (*p* ≤ 0.03) such as membrane and endoplasmic reticulum (Figure 3). For biological processes (*p* ≤ 0.03), the enriched terms included organelle organization, cell cycle and gene expression (Figure 3). For the WT versus *atg7*Δ analysis, 74 mRNAs were found enriched in the *atg7*Δ compared to the WT strain (Table S2). The most abundant cellular components mRNAs (*n* = 75) in *atg7*Δ EVs were the nucleus and the mitochondrion (Figure 4). Biological processes were associated to transcription, transcription regulation and RNA processing (Figure 4). However, the score values for some terms did not meet the statistics criteria (*p* ≤ 0.03).

Noteworthy, the second and third most abundant transcripts exclusively identified in *grasp*Δ EVs were those for the ER lumen protein retaining receptor and the regulator of vesicle transport through interaction with t-SNAREs 1 (Table S1). The former determines specificity of the luminal ER protein retention system and is required for normal vesicular traffic through the Golgi. The latter is involved in multiple transport pathways [34,35]. In addition, most of the transcripts were associated to organelles, such as the nucleus, the mitochondrion and the endoplasmic reticulum, suggesting that somehow the *GRASP* knockout resulted in altered population of transcripts composing the EVs. This enrichment profile was not observed in the *ATG7* knockout (Table S2), thus validating the differences observed in the *grasp*Δ mutant EVs.

**Figure 5:**
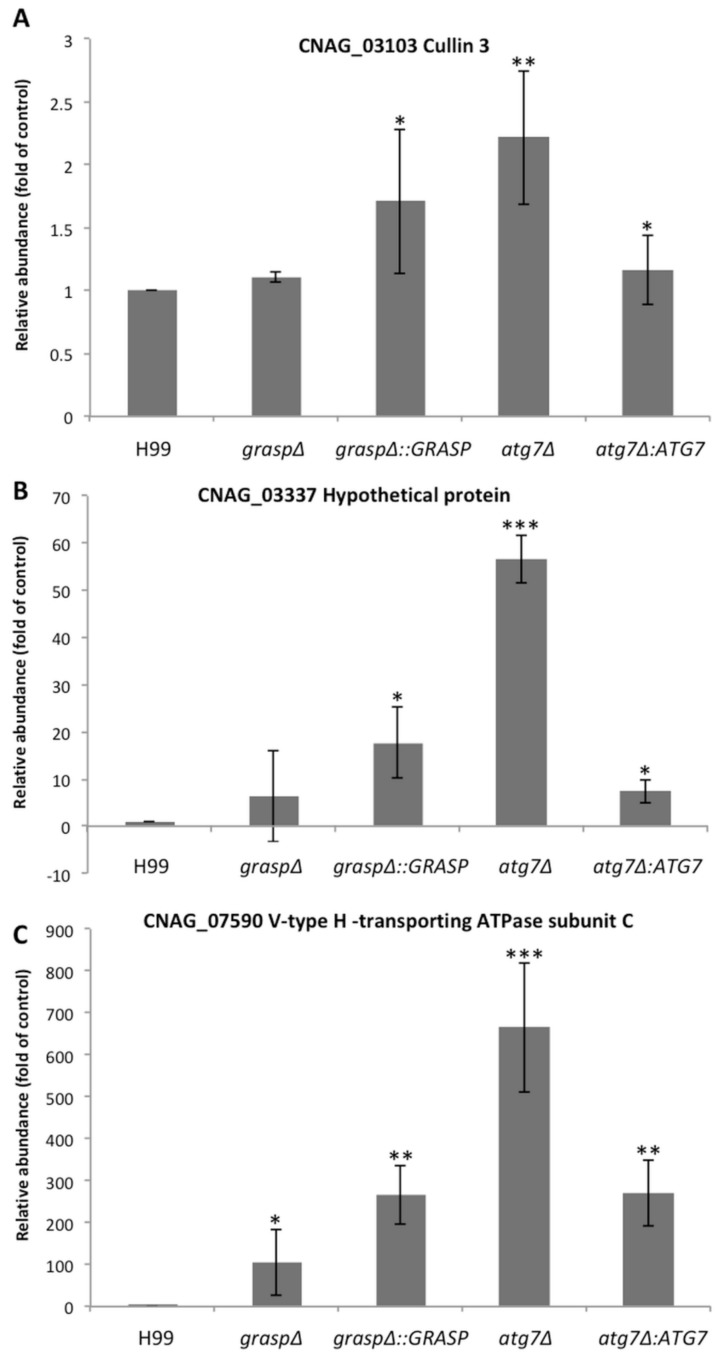
Analysis of the cellular transcription levels of three vesicular RNA sequences. Transcript levels for (**A**) Cullin 3; (**B**) hypothetical protein CNAG_03337 and (**C**) V-type H^+^-transporting ATPase subunit C were normalized to the levels of actin transcripts. The X-axis corresponds to each strain analyzed (WT, *grasp*Δ, *grasp*Δ::*GRASP*, *atg7*Δ and *atg7*Δ::*ATG7*). The *y*-axis corresponds to the relative expression level of the mRNAs in the cell. Each bar represents the mean and standard error of triplicate samples. * *p* < 0.05; ** *p* < 0.01; *** *p* < 0.001.

As we observed this alteration in the EV-RNA composition for the *grasp*Δ mutant we asked if this difference was due to a general alteration in the cell transcriptome caused by the *GRASP* knockout. We then selected three of the most enriched transcripts found in the *grasp*Δ mutant EVs and assessed their expression value by qPCR in WT, mutant (*grasp*Δ and *atg7*Δ) and complemented (*grasp*Δ::*GRASP* and *atg7*Δ::*ATG7*) strains (Figure 5). The expression values of cullin 3, hypothetical protein CNAG_03337 and the V-type H transporting ATPase subunit C transcripts were similar in WT and *grasp*Δ mutant strains, despite the mRNA alteration in the EVs obtained from these two strains. The *atg7*Δ mutant showed the highest expression levels of these mRNAs when compared to the WT and *grasp*Δ strains (Figure 5). In addition, these transcripts had very low identification or were not detected in EVs from the *atg7*Δ strain (Table S2). Therefore, despite of the fact that *atg7*Δ mutant showed high expression levels, this variation did not correlate with the presence of these transcripts in EV fractions. Analysis of the complemented strains demonstrated a partial restoration of the wild-type phenotype in the *atg7*Δ system (Figure 5). Altogether, these results reinforce the notion that the *GRASP* deletion lead to a shift in the RNA composition of cryptococcal EVs.

### 3.2. Comparison of Cellular RNA Versus Extracellular Vesicle RNA Composition

**Figure 6:**
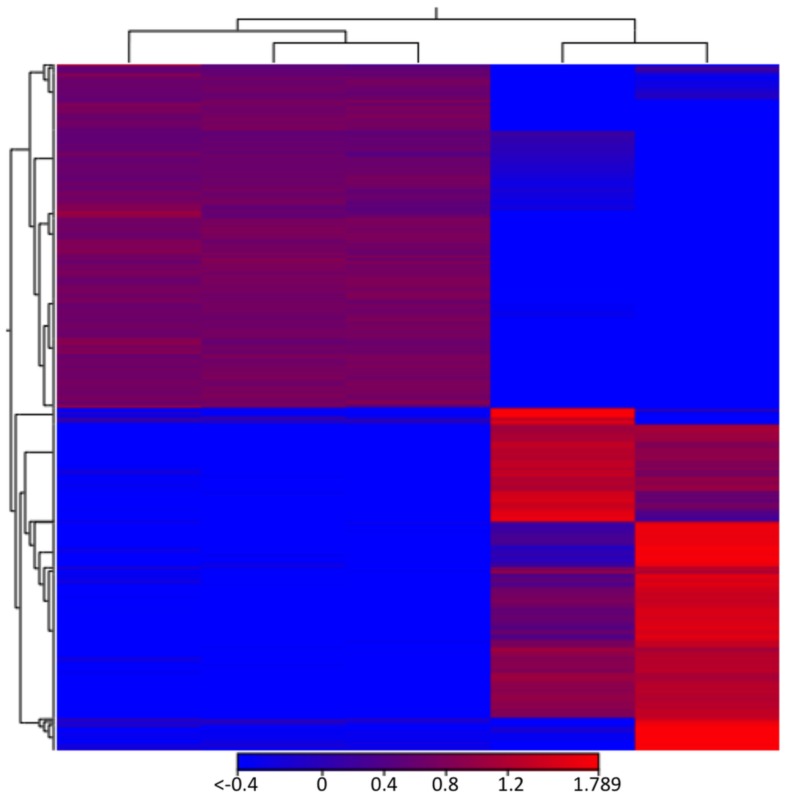
Heat map illustration of the comparison between cellular and EV RNAs. The expression levels are visualized using a gradient color scheme, where the red color is used for high expression levels and the blue color is used for low expression levels. Each line corresponds to a gene of the *C. neoformans* H99 strain.

The differences in the RNA composition of EVs produced by the *grasp*Δ strain led us to question whether the mRNAs in the EVs correspond to those highly expressed in the cell, likely resulting from random incorporation into vesicular carriers [36]. To address this hypothesis, we compared the *C. neoformans* transcriptome (H99 strain) with the vesicular RNA sequences [9,37] (Table S3). After applying the differential gene expression analysis (DGE) we observed that, for several transcripts, there was an inversion between the expression patterns in the cell and the RNA abundance in the EVs (Figure 6 and Table 2). For example, one of the most enriched transcripts in the EVs presented low levels of expression in the cell (CNAG_06651 amidohydrolase). On the other hand, CNAG_03012 (encoding a quorum sensing-like molecule) had an RPKM value greater than 20,000 in the cell but showed low abundance in the EVs (average RPKM value of 36; Table 3 and Table S3). This observation indicates a lack of correlation between the most expressed cellular mRNAs and EV cargo, therefore reinforcing the supposition that RNA loading into WT or mutant EVs is not random.

### 3.3. Intronic Reads

We have previously observed that a great number of *C. neoformans* EV-RNA reads mapped to intronic regions of the genome [9], which is in agreement with our current findings with the knockout strains. To analyze intronic reads in mRNAs and exclude non-coding RNAs (ncRNAs), such as ribosomal RNA (rRNA) or transfer RNA (tRNAs), we used the presence of exons as a criterion to ensure ncRNAs were excluded (Table 3). We observed two types of patterns, including reads mapping to both exons and introns in variable proportions and those that mapped only to introns in the messenger RNAs. The intronic mapping shared by the EV RNAs from the WT and mutant strains of *C. neoformans* were associated to translation and also to transmembrane proteins (Table 3). From the 32 mRNAs with intronic reads found in the EV samples, 12 have previously been described as transcripts with intron retention [38]. It has already been reported that 59% of the genes from *C. neoformans* use alternative splicing (AS) that varies depending on the growth conditions. The intron retention (IR) is the prevalent AS mechanism in this fungus [38]. We also observed differences in abundance between the cell mRNAs compared to those in the EVs (Table 3). For example, the mRNA CNAG_07982 that codes for a hypothetical protein is 10 times more abundant in the EV than in the cell. A similar profile was observed for sequence CNAG_01820, which encodes a pyruvate kinase (Table 3). It has been speculated that the mRNAs that present IR are not the most expressed in cells based on a negative correlation between the highly expressed transcripts and the presence of IR [38]. However, our present data show that most of the reads that were considered as aligned in introns, are in fact the rRNAs 25S, 18S and 5.8S (data not shown). Nevertheless, we obtained highly abundant transcripts that are likely to be intron-retaining mRNAs (Table 3), suggesting that somehow these IR mRNAs might be directed to the EVs. The function of these transcripts needs to be further investigated.

### 3.4. Non-Coding RNAs

The EV-RNA sequences obtained in this work also mapped to ncRNAs. The most abundant molecules were the 25S, 18S and 5.8S rRNAs, accounting for more than 90% of the ncRNA and intronic reads (data not shown). As described for the mRNA analysis, we performed paired comparisons (WT versus *grasp*Δ and WT vs. *atg7*Δ) and applied the statistical negative binomial test [33] and the filters RPKM ≥ 50, log2 ≥ 2 and FDR ≤ 0.01. For the WT versus *grasp*Δ we observed 43 ncRNAs enriched in *grasp*Δ (Table S4). For WT versus *atg7*Δ 30 ncRNAs were enriched in the *atg7*Δ strain (Table S5). From these results, it was possible to observe that the tRNA-derived fragments (tRFs) were enriched in both knockouts (Table S4 and S5). tRFs have been identified in EVs from organisms in all kingdoms, including archaea, bacteria and eukaryotes, where they play different biological roles [39].

## 4. Discussion

Fungal extracellular vesicles might correspond to structures that randomly incorporate cytosolic molecules that are released extracellularly or in the cell wall [11]. Our current results, however, suggest that EV RNA cargo can be finely regulated. Our model consisted of an investigation of the role of *C. neoformans* proteins GRASP and Atg

7 in the vesicular export of RNA. Although these proteins are functionally connected in other systems [12,17,26,27], our findings suggest that GRASP, but not Atg7, has a fundamental role in addressing RNA to cryptococcal EVs. The Atg proteins, which are primarily linked to autophagy processes, have non-canonical roles in distinct cellular pathways. It seems clear, however, that despite the variety of functions played by Atg7 and the significant alterations that its gene deletion causes in *C. neoformans*, the RNA populations transported by EVs were not greatly affected by the *atg7* knockout in *C. neoformans*. The phenotypic characteristics of this mutant included more efficient melanization, larger cell size, autophagic bodies formation and virulence attenuation [28].

Remarkably, phenotypic traits including EV dimensions were only partially recovered in complemented strains. This observation is likely related to methodological particularities intrinsic to the genetic manipulation of *C. neoformans*. For instance, biolistic transformation usually results in large chromosomic alterations but most importantly, gene complementation results in random insertion of *ATG7*- or *GRASP*-containing cassettes in multiple chromosome loci. Under these conditions, many phenotypic traits can be unpredictably affected and complemented genes can have their expression altered. In the specific case of *GRASP*, complementation of the *graspD* strain used in this study resulted in *GRASP* overexpression [20,28], which might be related to the unique phenotypic properties of the complemented strain.

Sequencing analysis of vesicular RNA obtained from mutant strains suggested that important biological functions are associated with nucleic acid-containing fungal vesicles. For example, the tRF-3’end derived (or CCA) uses the canonical miRNA machinery to downregulate replication of protein A1 mRNA and other transcripts in B cell lymphoma [40]. Regulation of translation is also a potential process where tRFs participate. It was demonstrated that tRF derived from tRNA-Val in the archaebacteria *Haloferax volcanii* binds to the small ribosomal subunit, consequently repressing translation by preventing a peptidyl transferase activity [41]. tRFs are also associated to the regulation of cell viability, RNA turnover and RNA stability [42,43,44,45]. The roles of GRASP and Atg7 in these processes have not been established but the enrichment of specific classes of RNA in mutant EVs suggests the existence of robust connections between EV traffic and tRFs. In *Trypanosoma cruzi*, the causing agent of Chagas disease, tRF-containing EVs can be transferred to other parasites and/or to host cells to modulate gene expression or facilitate infection [46,47]. In EVs from dendritic and T cells there are different populations of tRFs indicating selective loading of these molecules into the vesicles [48]. Human semen EVs are enriched with tRFs that hypothetically act as translational repressors [49]. It is unknown whether fungal vesicles can be transferred to other cells and consequently regulate metabolism and gene expression but it is tempting to speculate this hypothesis based on the findings mentioned above.

The mRNA population from *grasp*Δ EVs had low correlation with WT vesicles. In addition, ncRNA populations were also clearly distinct in EVs from WT and *grasp*Δ cells, where snoRNA predominated in the WT and tRNA/tRFs in the KOs.

The distinct RNA cargo in the mutants analyzed in this study is in agreement with a key and general role of GRASP in unconventional secretion in *C. neoformans* and a minor participation of Atg7. Polysaccharides, which lack secretory tags, require GRASP for efficient secretion in *C. neoformans* [20]. Deletion of *ATG7*, however, did not affect polysaccharide export in this fungus [28]. Multivesicular body formation and consequent exosome release involve a number of cellular regulators whose functions directly affect EVs [50,51]. In fungi, a number of regulators affect biogenesis of exosome-like EVs, including the ESCRT machinery, flippases and GRASP [52]. It has been hypothesized that GRASP (Grh1) could participate in this process by acting as a chaperone and directly influencing the cargo of EVs [53]. This GRASP chaperone function could be linked to our current results since RNA cargo was deeply affected in the *grasp*Δ mutant. Altogether, these results strongly indicate a novel function for the GRASP family in eukaryotes that could directly affect cell communication, gene expression and host-pathogen interactions.

## Figures and Tables

**Table 1:** RNA-seq mapping statistics. The values refer to the average of the replicates.

	*C. neoformans*					
	WT		*atg7*Δ		*grasp*Δ	
	Uniquely Mapped	% of Total Mapped	Uniquely Mapped	% of Total Mapped	Uniquely Mapped	% of Total Mapped
Exon	5030	0.4	60,683	9.2	59,425	7.5
Exon-exon	10,664	0.6	1458	0.2	2350	0.3
Total exon	113,655	9.7	62,141	9.4	61,774	7.8
Total intron	1,003,971	90.3	568,003	84.9	758,109	86.9
Total gene	1,117,625	100	667,288	100.0	861,092	100.0

**Table 2:** Comparison between cellular and vesicular RNA in *C. neoformans* (H99 strain). The top ten most expressed transcripts in the cell are shown in light blue. The most represented RNAs in the EVs are illustrated in light red.

Name	Product	EV vs. Cell-Log Fold Change	EV vs. Cell-FDR *p*-Value	SRR3199612 Cell 1 RPKM	SRR3199613 Cell 2-RPKM	SRR3199614Cell 3-RPKM	EV RNA 1-RPKM	EV RNA 2-RPKM
CNAG_03012	quorum sensing-like molecule	−5.06	0.00%	20,332.13	18,844.93	20,155.19	48.40	24.28
CNAG_06207	hypothetical protein	−6.93	0.00%	16,304.37	14,037.19	13,815.62	8.35	6.02
CNAG_04105	hypothetical protein	−2.31	2.29%	16,003.01	10,010.74	16,467.89	254.98	73.62
CNAG_03143	hypothetical protein	−2.09	3.87%	13,070.91	8338.53	12,401.56	231.90	78.39
CNAG_01735	hypothetical protein	−3.60	0.03%	9034.37	6373.02	7970.58	56.97	16.11
CNAG_06075	hypothetical protein	−2.98	0.39%	6021.45	5119.83	6051.19	66.75	13.02
CNAG_03007	hypothetical protein	−6.58	0.00%	5861.25	5356.39	4321.23	3.72	2.64
CNAG_06298	hypothetical protein	−7.02	0.00%	5319.83	5499.72	6635.36	2.65	3.96
CNAG_06101	ADP, ATP carrier protein	−3.04	0.11%	4475.82	5415.80	4535.39	44.21	32.06
CNAG_07466	U3 small nucleolar RNA-associated protein 7, U3 small nucleolar RNA-associated protein 7, variant 1, U3 small nucleolar RNA-associated protein 7, variant 2	10.05	0.00%	392.25	765.29	487.61	39,204.58	43,212.28
CNAG_01093	hypothetical protein	8.04	0.00%	45.01	41.14	33.25	831.32	480.72
CNAG_06651	amidohydrolase	12.70	0.00%	3.80	4.64	3.38	777.33	4354.12
CNAG_00311	3-hydroxyisobutyryl-CoA hydrolase	6.97	0.00%	62.61	78.16	54.48	650.07	373.07
CNAG_02129	hypothetical protein	2.12	3.76%	423.90	380.09	587.72	178.91	56.55
CNAG_05774	hypothetical protein, hypothetical protein, variant	4.48	0.00%	87.07	87.32	82.46	146.53	104.07
CNAG_05651	hypothetical protein	7.83	0.00%	5.28	8.41	7.53	138.73	51.57
CNAG_07515	hypothetical protein	4.79	0.00%	57.55	52.26	77.35	118.53	127.65
CNAG_04124	hypothetical protein	7.66	0.00%	6.60	6.26	5.59	113.90	21.83
CNAG_07028	26S proteasome regulatory subunit N11	4.03	0.00%	103.45	112.54	86.34	112.59	118.66

**Table 3:** Intron retention in EV RNAs.

ID	Data obtained from Gonzalez-Hilarion et al., 2016 [38]				RPKM	Unique Exon Reads	Unique Intron Reads	RPKM	Unique Exon Reads	Unique Intron Reads	RPKM	Unique Exon Reads	Unique Intron Reads	Product
	intron	Type	RPKM	Exons	WT			Δ*Atg7*			Δ*GRASP*			
CNAG_03602	Ic2-554	in5UTR	**6.66**	5	**23.5**	4	228	**40.59**	3	87.5	**28.57**	1.5	81	U3 small nucleolar RNA-associated protein 5
	Ic2-555	in5UTR	**204.80**											
CNAG_03645	Ic2-787	inCDS	**8.01**	8	**6.3**	1.5	73.5	**53.43**	5.5	9.5	**58.98**	6.5	14.5	NET1-associated nuclear protein 1 (U3 small nucleolar RNA-associated protein 17)
	Ic2-788	inCDS	**12.93**											
CNAG_04068	Ic2-3155	inCDS	**3.18**	4	**71.2**	3.5	10	**270.93**	6	11	**522.07**	12	4.5	large subunit ribosomal protein L28e
CNAG_07982	Ic4-247	inCDS	**6.96**	5	**1061.8**	60.5	100.5	**81.42**	5	3	**60.25**	3.5	7	hypothetical protein
	Ic4-248	inCDS	**17.47**											
CNAG_00930	Ic4-351	inCDS	**2.61**	7	**50.1**	5.5	328	**81.47**	4	303.5	**106.30**	6	397	argininosuccinate synthase
	Ic4-349	in5UTR	**1066.15**											
	Ic4-350	in5UTR	**81.59**											
CNAG_07884	Ic8-1359	inCDS	**10.84**	3	**7.4**	0.5	66	**18.73**	0.5	21.5	**85.27**	2.5	21	hypothetical protein
CNAG_07813	Ic12-778	inCDS	**8.46**	5	**75.4**	6.5	231.5	**14.71**	0.5	141.5	**37.66**	1	208.5	hypothetical protein
	Ic12-776	in5UTR	**7.38**											
CNAG_06167	Ic13-990	in5UTR	**26.36**	5	**131.1**	11	770	**103.14**	6.5	152.5	**146.05**	10.5	295	metal homeostatis protein bsd2
CNAG_01820	Ic3-1947	in3UTR	**28.14**	12	**342.4**	47	180.5	**236.94**	17	15.5	**344.85**	27.5	15	pyruvate kinase, pyruvate kinase, variant
CNAG_06033	Ic13-230	inCDS	**10.93**	7	**99.4**	15	22	**47.14**	3.5	5	**33.92**	3.5	63.5	pfkB family carbohydrate kinase superfamily
CNAG_03730	Ic2-1335	in5UTR	**35.87**	4	**41.7**	2	479.5	**102.24**	2	23.5	**0.00**	0	175.5	DNA-directed RNA polymerase II subunit RPB11
CNAG_06401	Ic14-772	in5UTR	**14.61**	11	**6.1**	0.5	23	**52.25**	4.5	162.5	**28.12**	2.5	27.5	hypothetical protein

## Supplementary Materials

The following are available online at http://www.mdpi.com/2073-4425/9/8/400/s1, Supplementary Table S1: List of transcripts enriched in *grasp*Δ compared to the wild type strain (H99), Supplementary Table S2: List of transcripts enriched in *atg7*Δ compared to the wild type strain (H99), Supplementary Table S3: List of transcripts enriched in the EVs compared to the cell in the wild type strain H99, Supplementary Table S4: List of ncRNAs enriched in *grasp*Δ compared to the wild type strain (H99), Supplementary Table S5: List of ncRNAs enriched in *atg7*Δ compared to the wild type strain (H99), Supplementary Figure S1: Electropherograms of the small-RNA content of EVs from the WT, *grasp*Δ and *atg7*Δ strains of *C. neoformans*. The size in nucleotides (nt) and the fluorescence intensity (FU) are indicated on the corresponding axes of the graphs generated from the profiles shown on the left.
