# Supplementary figures and images for: Golgi Reassembly and Stacking Protein (GRASP) Participates in Vesicle-Mediated RNA Export in Cryptococcus neoformans
Source: Genes (Basel). 2018 Aug 8;9(8):400. doi: 10.3390/genes9080400 (PMC6115741; doi:10.3390/genes9080400)

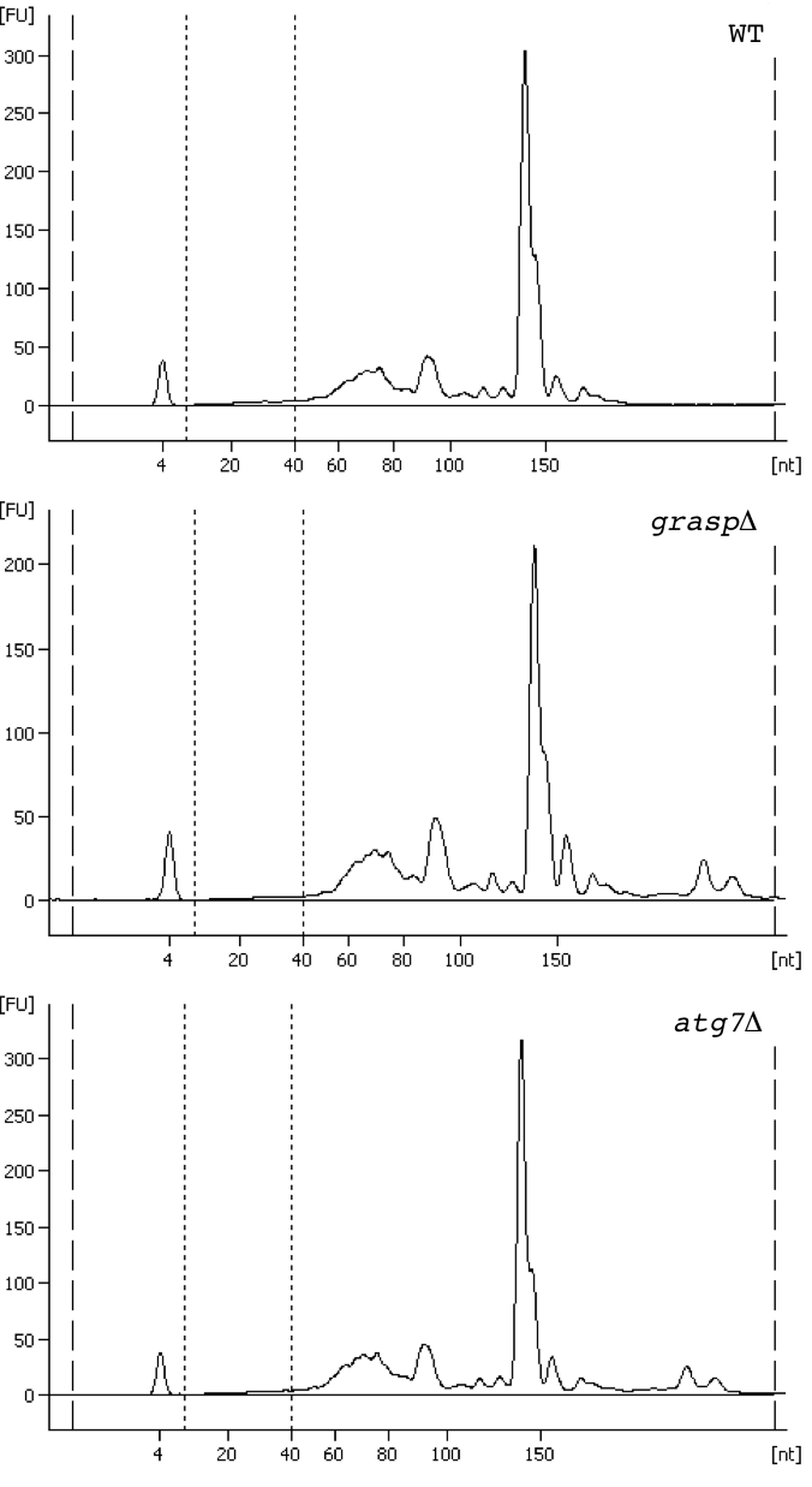

Supplement: Supplementary file 1 [file genes-09-00400-s001.zip › genes-329952-SI/Figure S1.tif]
